# Identification of Astrocytoma Blood Serum Protein Profile

**DOI:** 10.3390/cells9010016

**Published:** 2019-12-19

**Authors:** Paulina Vaitkiene, Ruta Urbanaviciute, Povilas Grigas, Giedrius Steponaitis, Arimantas Tamasauskas, Daina Skiriutė

**Affiliations:** 1 Laboratory of Molecular Neurooncology, Neuroscience Institute, Lithuanian University of Health Sciences, Eiveniu str. 4, LT50161 Kaunas, Lithuania; ruta.urbanaviciute@lsmuni.lt (R.U.); giedrius.steponaitis@lsmuni.lt (G.S.); arimantas.tamasauskas@lsmuni.lt (A.T.); dainski@gmail.com (D.S.); 2 Laser Research Centre (VU LRC), Vilnius University, Sauletekio Ave 10, LT10223 Vilnius, Lithuania; pgrigas@gmail.com

**Keywords:** astrocytoma, glioblastoma, serum proteins, Protein Antibody Array, decision tree, survival

## Abstract

High-grade astrocytomas are some of the most common and aggressive brain cancers, whose signs and symptoms are initially non-specific. Up to the present date, there are no diagnostic tools to observe the early onset of the disease. Here, we analyzed the combination of blood serum proteins, which may play key roles in the tumorigenesis and the progression of glial tumors. Fifty-nine astrocytoma patients and 43 control serums were analyzed using Custom Human Protein Antibody Arrays, including ten targets: ANGPT1, AREG, IGF1, IP10, MMP2, NCAM1, OPN, PAI1, TGFβ1, and TIMP1. The decision tree analysis indicates that serums ANGPT1, TIMP1, IP10, and TGFβ1 are promising combinations of targets for glioma diagnostic applications. The accuracy of the decision tree algorithm was 73.5% (75/102), which correctly classified 79.7% (47/59) astrocytomas and 65.1% (28/43) healthy controls. The analysis revealed that the relative value of osteopontin (OPN) protein level alone predicted the 12-month survival of glioblastoma (GBM) patients with the specificity of 84%, while the inclusion of the IP10 protein increased model predictability to 92.3%. In conclusion, the serum protein profiles of ANGPT1, TIMP1, IP10, and TGFβ1 were associated with the presence of astrocytoma independent of its malignancy grade, while OPN and IP10 were associated with GBM patient survival.

## 1. Introduction

High-grade astrocytomas, especially glioblastomas (GBMs), are one of the most aggressive brain tumors, with a poor prognosis. Generally, brain astrocytomas are diagnosed only after the occurrence of clinical symptoms, and a diagnosis is determined only after the histological examination of the tissue [1]. However, histological data and clinical symptoms fail to explain variation in astrocytoma progression and the response to treatment. There is an increasing interest in identifying molecular markers that better capture the status of a tumor to improve the existing predictions. Especially, there is a need of biomarkers that can indicate a change in protein expression which are related to the risk or progression of astrocytoma or with the response to a given treatment, survival, or recurrence [2,3]. Recently, gliomas could be subtyped based on several molecular markers like IDH1, 1p/19q co-deletion, EGFR amplification, loss of PTEN, MGMT methylation, etc., to predict patient prognosis while considering parameters like patient age and complete tumor histopathological profile [4]. Minimally invasive and early diagnostic techniques could play an important role in adapting the best treatment for astrocytoma patients [5]. A great effort is currently being made to achieve methods for early tumor detection, especially including methods based on the detection of proteomic profiles in serum/plasma. A good alternative astrocytoma screening test should be as minimally invasive as possible, achieve excellent analytical outcomes regarding sensitivity and specificity, and still be fast and cost-effective. An optimal test could help to monitor if changes in the composition of serum proteins could indicate astrocytoma occurrence or recurrence and also help to optimize a treatment strategy. Therefore, the field of intensive research is moving toward extensive serum proteome profiling for tumor-specific markers [2]. Furthermore, the determination of a tumor-specific panel of markers could outperform the evaluation of individual markers regarding specificity and sensitivity. Moreover, multiple-protein profiling could provide a more detailed image of molecular networks and pathophysiological conditions of the disease. The determination of multiple proteins from a single sample could be done by using biochip array technology [6]. Biochip array technology is also practicable when volumes of clinical samples are limited.

Astrocytic glioma screening for the application of serological testing has not been established so far, even though very promising candidate markers have been reported based on proteomic [7,8,9] and individual target screenings [1,2,3]. Our study aimed to characterize preoperative protein levels in the blood serum of patients with different malignancy grade astrocytoma compared with healthy subjects. We focused on already-known cancer-related proteins based on a thorough literature review [1,3,6,10,11,12,13,14,15,16,17,18,19,20,21,22] and our earlier laboratory findings [23,24]. The ten proteins which were compatible to be analyzed in a protein multiplex array manner selected for the analysis were: ANGPT1 (angiopoietin-1), AREG (amphiregulin), IGF1 (insulin like growth factor I), IP10 (CXCL10, C-X-C motif chemokine 10), MMP2 (matrix metallopeptidase 2), NCAM1 (neural cell adhesion molecule 1), OPN (osteopontin), PAI1 (plasminogen activator inhibitor 1), TGFβ1 (transforming growth factor beta 1), and TIMP1 (TIMP metallopeptidase inhibitor 1). The novelty of the study was the simultaneous assessment of ten serum proteins using a multiplex biochip array to identify a clinically applicable combination of serum markers for glioma screening. This study revealed the possibility of using a multiplex immunoassay to screen for novel and valuable blood-based protein biomarkers for malignant astrocytomas. 

## 2. Materials and Methods

### 2.1. Study Group

This study was comprised of 102 serum samples that were collected at the Hospital of Lithuanian University of Health Sciences neurosurgery clinics (Kaunas, Lithuania) from 2015 to 2017. The study was carried out under the Ethical Principles for Medical Research Involving Human Subjects (Declaration of Helsinki) and the research methodologies were approved by the Kaunas Regional Biomedical Research Ethics Committee (No. P2-9/2003). All subjects gave their informed consent for inclusion before they participated in the study. We investigated 39 patients with histopathologically confirmed glioblastoma (grade IV), 3 patients with anaplastic astrocytoma (grade III), 17 patients with diffuse astrocytoma (grade II), and 43 healthy controls. The control group was age-adjusted to the case group (median/mean age for patient group 49.82/49.59 years; median/mean age for control group 44.21/45.01 years) (*t*-test, *p* > 0.05). For astrocytoma patients, blood was taken before the operation and any kind of treatment (e.g., chemotherapy or radiotherapy). Serum samples were prepared as follows: blood samples were allowed to clot for 30 min at room temperature before centrifuging for 15 min at 1000× *g*. Serum was removed, aliquoted, and samples were stored at −70 °C. Data on patient age at the time of the operation and gender were collected. None of the patients had received chemotherapy or radiation before surgery, while after surgery they had received the standard treatment. Patients’ overall survival was computed from the date of the operation to the date of the death or the date of the survey database closure (censored). The database closure was in January 2018. The date and cause of death were obtained from The Lithuanian State Register of Death Cases.

### 2.2. Evaluation of Serum Proteins Level

For simultaneous quantitative detection of multiple proteins from a single patient sample we used Custom Human Antibody Array Membranes (ten targets) (Ab211921, Abcam, Cambridge, UK). The biochip comprised printed capture antibodies against ANGPT1, AREG, IGF1, IP10, MMP2, NCAM1, OPN, PAI1, TGFβ1, and TIMP1. The protein array is an antibody-pair-based assay which performs analogously to ELISA but using a membrane as a substrate rather than a plate, as capture antibodies are spotted on a membrane. The assay was performed following the manufacturer’s instructions with some modifications. A total of 1 mL of 5× diluted blood serum was used for each reaction. After incubation, a Biotinylated 10 Antibody Cocktail (horseradish peroxidase (HRP)-labeled secondary antibodies) was added to the membranes. After following washes to remove unbound material, protein levels were quantified by chemiluminescence using the BioSpectrum Imaging System (UVP, Analytik Jena Company, Cambridge, UK). The signal intensity of each antigen-specific antibody spot represents a relative proportion of the antigen amount in the sample. Three control spots on every array were used: positive, negative, and blank control spots. The Positive Control (PC) spots (six spots per array), which contained biotin-conjugated IgG, were used for signal normalization. Negative Control (NC) (four spots per array) printed with buffer and blank spots (two spots per array) were used to determine nonspecific binding and for background subtraction. For the signal evaluation, the image processing program ImageJ was applied. After obtaining raw densitometry values, the intensity for every duplicated spot was averaged and normalized, subtracting mean intensity of the background (NC). Averaged PC spots were used for intra-array normalization. For inter-array normalization, all the membranes were adjusted to the reference array. The signal intensity of every spot representing different analyte was calculated as follows: *X(Ny) = X(y) ∗ P1/P(y)*, where: P1—averaged signal density of PC spots on reference array; P(y)—averaged signal density of PC spots on membrane y; X(y)—averaged signal intensity of analyte X on membrane y; X(Ny)—normalized signal intensity for spot X on membrane y. For the reduction of technical variation and more accurate appreciation of biological differences, the relative protein level was calculated and used for further statistical analysis.

### 2.3. Statistical Analysis

For statistical analysis, the software package SPSS Statistics 19 (SPSS Inc., Chicago, IL, USA) and GraphPad Prism (GraphPad Software, La Jolla, CA, USA) were used. Chi-square test, linear regression analysis, Spearman’s correlation coefficient, Kruskal–Wallis (comparison of >2 groups) and Mann–Whitney tests were used to analyze data. Decision tree classification was performed using R Software version 3.3.3, rpart package. The classification was carried out applying binary classification where RSS (ANOVA) selected as a split criterion. In total 100 random tree models were created at learning stage to ensemble method for classification. The random tree models test divided the dataset randomly into learning (approximately 60% of the data set) and test (approximately 40% of the data set) subsets. When we generated 100 tree models, the dominant model was chosen and used further. At the end of the process, the errors were counted from this dominant model. The Kaplan–Meier method was used to estimate survival functions. A significance level of 0.05 was selected to test the statistical hypothesis.

## 3. Results

### 3.1. Serum Analysis of Single Candidate Proteins

**Figure 1:**
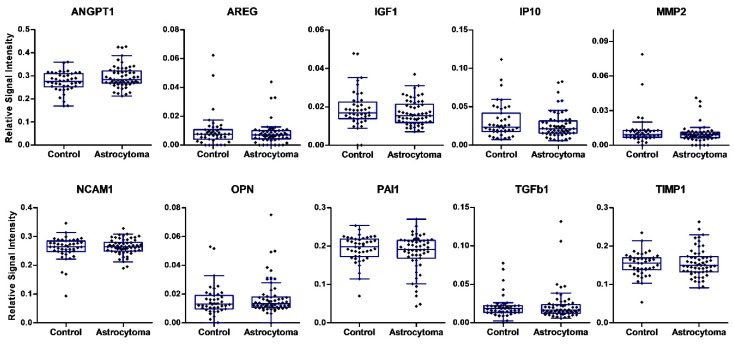
Proteomic analysis of ten proteins in preoperative blood serum in astrocytoma patients and controls. Box plots of relative expression measurements of proteins ANGPT1, AREG, IGF1, IP10, MMP2, NCAM1, OPN, PAI1, TGFβ1, and TIMP1 obtained by protein array analysis of different malignancy grade astrocytoma patients’ serum and normal controls. The line inside each box represents the median; the lower and upper edges of the boxes represent the 25th (first quartile) and 75th (third quartile) percentiles, respectively; the upper and lower edges of the boxes represent the Tukey’s whiskers.

The expressions of ten proteins in peripheral blood serum specimens were analyzed in our study using an ELISA-based multiplex protein array. Serum samples were collected prior to brain surgery from 59 patients with different-grade astrocytoma and 43 controls. The expression level of ten serum proteins was quantified and compared among groups (Figure 1). Comparisons of box-and-whiskers plots for every analyte measurement in the serum of astrocytoma patients and control samples revealed consistent distribution and median levels. No significant differences in the levels of single proteins were observed between the normal group and astrocytoma patients. 

### 3.2. Combined Analysis of Protein Levels in Astrocytoma Serum

The results of ten differentially expressed proteins on the array were used to construct a decision tree classification algorithm, in an attempt to identify potential serum biomarkers for astrocytoma. 

**Figure 2:**
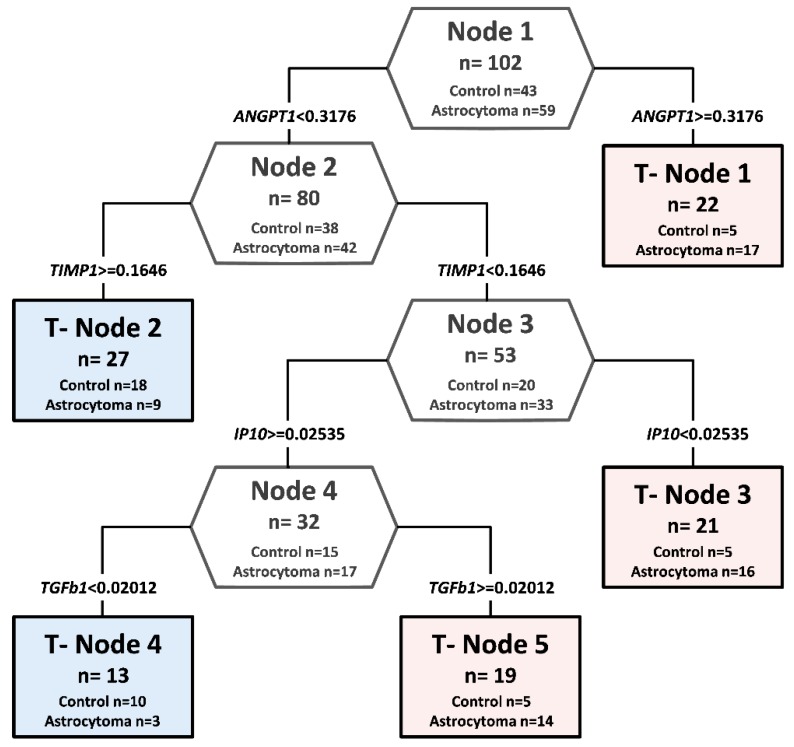
Decision tree classification diagram of protein relative values from astrocytoma patients and controls, using protein microarrays. Potential diagnostic serum protein profile composed of ANGPT1, TIMP1, IP10, and TGFβ1. The numbers in the root node (top), descendant nodes (hexagons), and terminal nodes (rectangles) represent the classes (astrocytoma patients and controls, *n* represents the sum of astrocytoma patients and controls). The number below the root and descendant nodes indicate the relative values of protein.

In a decision tree analysis, relative expression level values of ten candidate proteins in 59 different grade astrocytoma patients’ serum was compared with protein relative expression values in 43 healthy controls. Of ten analyzed proteins, statistically different values of four proteins were chosen as the predictor to build up the decision classification tree shown in Figure 2. The classification algorithm used values of four proteins, and five terminal nodes were determined with the relative cost of 0.27. ANGPT1, TIMP1, IP10, and TGFβ1 together in the complex were identified as the most promising potentially diagnostic serum profile. The accuracy of the decision tree algorithm was 73.5% (75/102), which correctly classified 47 of 59 astrocytomas (79.7%) and 28 of 43 healthy controls (65.1%). 

### 3.3. Survival Analysis

**Figure 3:**
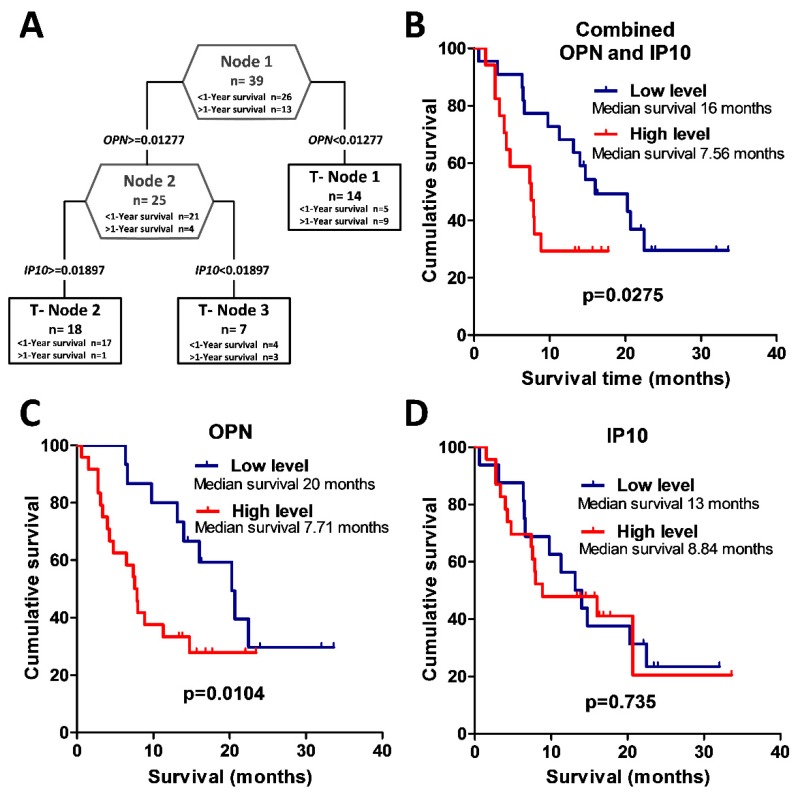
Potential prognostic serum protein profile for glioblastoma patients composed of OPN and IP10. (**A**) Glioblastoma patient decision tree with the clinical feature of one-year post-surgery survival. The numbers in the root node (top), descendant nodes (hexagons), and terminal nodes (rectangles) represent the classes (glioblastoma patients who survived less than one year after surgery and glioblastoma patients who survived more than one year after surgery); *n* = sum of glioblastoma patients in a class. The numbers below the root and descendant nodes indicate the relative values of protein. (**B**–**D**) Kaplan–Meier survival curves. (**B**) Kaplan–Meier analysis of glioblastoma patient overall survival differences between combined OPN and IP10 protein expression groups. Low- vs. high-level protein patient group median survival 16 months versus 7.56 months (log-rank test, *p* = 0.0275). (**C**) Kaplan–Meier analysis of glioblastoma patient overall survival between OPN protein relative expression groups, were OPN low level means that relative OPN protein value is <0.01277, and OPN high level means that relative OPN protein value is ≥0.01277. Low- vs. high-level protein patient group median survival 20 months versus 7.71 months (log-rank test, *p* = 0.0104). (**D**) Kaplan–Meier analysis of glioblastoma patient overall survival in different IP10 protein relative expression groups, where IP10 low level means that relative IP10 protein value is <0.01897, and IP10 high level means the value is ≥0.01897. Low- vs. high-level protein patient group median survival 13 months versus 8.84 months (log-rank test, *p* = 0.735).

The GBM group was subdivided into two groups comprising patients who survived post surgery for more than one year (*n* = 26) and those who survived for less than one year (*n* = 13). For the clinical feature of post-surgery one-year survival, an association was achieved with serum profiles based on relative concentrations of OPN and IP10 (Figure 3A). Analysis of OPN protein relative value alone predicts the survival of more than one year with a specificity of 84% in GBM patients, while the inclusion of the other factor (IP10 protein relative value) increased specificity to 92.3%. The two survival groups were not distinguished by different treatment schemes because standard therapy, including surgery, radio, and chemotherapy, were used for both groups. The Kaplan–Meier analysis using the log-rank test showed an association between GBM patient overall survival and relative OPN expression groups (log-rank test, χ^2^ = 3.95, df = 1, *p* = 0.047) (see Figure 3C). Glioblastoma patients with low serum OPN expression had a significantly higher chance for longer survival when compared to patients having high serum OPN expression. Kaplan–Meier analysis showed that there was no significant difference in overall survival comparing all glioblastoma patients with relatively high or low IP10 expression (log-rank test, χ^2^ = 0.03, df = 1, *p* = 0.869) (see Figure 3D). Further, we aimed to identify the combination of protein expression values that could help to prognosticate patient survival after surgery. 

Therefore, to analyze glioblastoma patient survival, patients were divided into two groups. One group included glioblastoma patients who had the following relative expression value of protein OPN < 0.01277 and OPN ≥ 0.01277, but IP10 < 0.01897. The other group consisted of glioblastoma patients with relative values of OPN ≥ 0.01277 and IP10 ≥ 0.01897. The Kaplan–Meier analysis using the log-rank test showed a significant association between glioblastoma patient overall survival and combined OPN and IP10 protein expression groups (log-rank test, χ^2^ = 4.04 df = 1, *p* = 0.044) (see Figure 3B). 

## 4. Discussion

This study revealed the possibility of using multiplex immunoassays to screen for novel and valuable blood-based protein biomarkers for malignant astrocytoma. Both unique and prominent protein candidates associated with malignant astrocytoma were studied using multiplex protein immunoassays with serum samples. 

In addition, protein biomarker candidates associated with malignant astrocytoma prognosis were included in this analysis. The aim was to characterize the preoperative levels of ten proteins in the blood serum of patients with different malignancy grades of astrocytoma and to compare them with healthy controls. The novelty of the study was the simultaneous assessment of ten serum proteins using multiplex biochip arrays to identify a clinically applicable combination of serum markers for glioma screening. Our decision was to focus on already-known cancer related proteins due to numerous published data. Based on a thorough literature review [1,3,6,10,11,12,13,14,15,16,17,18,19,20,21,22] and our laboratory findings [23,24], the ten proteins which were compatible to be analyzed in a protein multiplex array manner selected for the analysis were: ANGPT1, AREG, IGF1, IP10, MMP2, NCAM1, OPN, PAI1, TGFβ1, and TIMP1. Many markers included in our multiplex biochip array have been thoroughly investigated individually in other researchers’ previous studies. In our study, no significant differences in single protein levels between gliomas and healthy controls’ serum were established. This could be related to the relatively small sample size in our study or the differences in the methodologies compared to other studies. Additionally, the variability in blood serum protein levels could be related to the size of the tumor and might prevent the establishment of precise protein level cutoff values for cancer diagnosis, considering large personal variations among patients—at least when using single protein biomarkers. Single protein markers in serum are mostly inadequate to follow the dynamics of glioma. This could be improved by using multiple marker profiles. In this study, we tried to search for a diagnostic serum profile in astrocytoma patients, using a multiplex protein array and target combination approach. When tested in serum, none of our chosen single proteins was sufficiently specific to serve as a diagnostic marker. Data showed that in contrast to single biomarkers, in a complex diagnostic profile of multiple proteins it may not be necessary for the concentrations of each protein to be significantly different between two groups [1]. Our results support these findings. Using the multiplex ten protein array and decision tree analysis, we found combinations of serum proteins with significant differences in protein levels between astrocytomas and healthy controls’ serum. A profile with a relatively small number of proteins (ANGPT1, TIMP1, IP10, TGFβ1) was sufficient to correctly assign 79.7% (47/59) of the astrocytoma and 65.1% (28/43) of the control subjects. Astrocytoma progression is related to rapid tumor growth and invasiveness. Astrocytomas express growth factors and receptors that are typically induced during angiogenesis; one of them is angiopoietin-1 (ANGPT1) [16]. During the development of the immature vasculature, ANGPT1 is critically involved in angiogenesis, and influences processes such as remodeling, maturation, and maintenance of the vascular plexus [16]. Depending on its function, ANGPT1 could be a molecular marker of glioma malignancy. Hands and colleagues, using a BioPlex immunoassay to provide cytokine and angiogenesis factor levels that differ between serum from glioma and non-cancer patients, found angiopoietin as the one important molecular marker [17]. In our study, ANGPT1 was one of the main serum proteins that helped to distinguish healthy subjects from glioma patients. TIMP1, a natural inhibitor of matrix metalloproteinase (MMP) enzymes, inhibits in vitro and in vivo tumor cell invasion and binds several members of the metalloproteinase family [18]. Crocker and colleagues’ data suggest that, in glioblastoma patients, a higher serum TIMP1 level at tumor presentation predicted shorter survival and that TIMP1 levels in the glioblastoma patients are significantly higher than in normal controls [6]. We found TIMP1 as one of the proteins which help to distinguish healthy from gliomas, but in our study, its significance for survival was not confirmed. Schneider et al. analyzed plasma from 21 patients with glioblastoma before and after resection using the ELISA method and found that concentrations of latent TGFβ1 of patients with glioblastoma before surgery were significantly higher in comparison to healthy control probands [19]. They also found a weak relation between long survival and a low concentration of the latent form of TGF-β1 prior to surgery [19]. We identified that relative TGFβ1 protein levels in astrocytoma patient serum samples before surgery may help in evaluating protein profiles. Our proposed algorithm includes IP10 protein values. It should be noted that these are in line with the data of Elstner and colleagues, where using conventional ELISA, reduced serum concentrations of IP10 were found in GBM patients compared to controls [1]. In our proposed algorithm, low IP10 protein values helped to identify astrocytoma patients. Sreekanthreddy et al. found that OPN expression in tissue was upregulated in GBM, and elevated serum OPN levels in GBM patients were also shown [13]. GBM patients with high serum OPN levels had poorer survival rates than those with low serum OPN levels [13]. Our study has shown that individually protein OPN was statistically and reliably associated with GBM survival, but after decision tree analysis, the specificity of the study could be improved by entering in the model protein IP10 relative value. On the other hand, single IP10 protein value was not significantly related to patient survival. This reaffirms the claim that in contrast to single biomarkers, in a complex prognostic protein profile it may not be necessary that each protein value be significantly different between two groups. 

## 5. Conclusions

Our data suggest an algorithm for the indication of the presence of astrocytoma-specific serum profiles. Prospective and retrospective validation of the data on a larger sample would be necessary to evaluate the algorithm and perhaps adjust any other proteins, which would improve the accuracy of the method. Glioblastoma detection, applying the analysis of blood serum, could yield multiple benefits, including the early intervention of therapy, reduction in mortality and morbidity, as well as monitoring the effect of therapy.
